# Personality Traits, Affective Distress, and Addictive Behaviors in Patients with Neurotic Disorders: A Mediation Analysis

**DOI:** 10.3390/ejihpe16030035

**Published:** 2026-03-04

**Authors:** Marin Mamić, Goranka Radmilović, Jakov Ivković, Bruno Dokozić, Danijel Mikulić, Ivana Mamić, Valentina Matijević, Ivan Vukoja

**Affiliations:** 1 General County Hospital Požega, Osječka 107, 34000 Požega, Croatia; goranka.radmilovic@pozeska-bolnica.hr (G.R.); bruno.dokozic@pozeska-bolnica.hr (B.D.); ivana.mamic@pozeska-bolnica.hr (I.M.); ivan.vukoja@pozeska-bolnica.hr (I.V.); 2 Faculty of Dental Medicine and Health Osijek, Josip Juraj Strossmayer University of Osijek, Crkvena 21, 31000 Osijek, Croatia; 3 Faculty of Medicine, Josip Juraj Strossmayer University of Osijek, J. Huttlera 4, 31000 Osijek, Croatia; 4 Naftalan Special Hospital for Medical Rehabilitation, Omladinska 21, 10310 Ivanić-Grad, Croatia; jakov.ivkovic@naftalan.hr; 5 University Hospital Center Sestre Milosrdnice, Vinogradska cesta 29, 10000 Zagreb, Croatia; danijel.mikulic@kbcsm.hr; 6 School of Medicine, Catholic University of Croatia, Ilica 242, 10000 Zagreb, Croatia

**Keywords:** personality traits, neuroticism, anxiety, depression, alcohol addiction, nicotine addiction, mediation analysis, neurotic disorders

## Abstract

This study investigated an integrative mediation model examining whether anxiety and depression mediate the relationship between the Big Five personality traits and the severity of alcohol and nicotine dependence among psychiatric patients with neurotic disorders (ICD-10 codes F40–F48). A cross-sectional design was conducted on a clinical sample of 232 patients (57.3 female; mean age = 48.58, SD = 10.77) using standardized instruments: Big Five Inventory (BFI-44), Fagerström Test for Nicotine Dependence (FTND), Michigan Alcoholism Screening Test (MAST), and Depression, Anxiety, and Stress Scale (DASS-21). Data were analyzed using MLR mediation modeling. The model explained 32.6 of the variance in nicotine dependence and 27.1 in alcohol dependence. Results revealed a pattern of complete mediation: neuroticism had no direct effect on addiction but influenced alcohol dependence exclusively through anxiety (*p* = 0.001) and nicotine dependence through depressive symptoms (*p* = 0.012). Extraversion and agreeableness showed a dual role, exerting significant direct positive paths toward addiction severity (*p* = 0.005) while simultaneously reducing it through negative indirect effects on affective distress. Overall, neuroticism was confirmed as a universal risk factor for mental health issues. These findings suggest that personality-driven addiction in neurotic patients is operationalized through specific clinical symptoms, highlighting the necessity for therapeutic interventions focused on targeted affect regulation and social assertiveness to mitigate substance use in this population.

## 1. Introduction

Depression and anxiety are among the leading causes of disability, morbidity, and mortality worldwide and are particularly prevalent among people with disorders caused by various addictions (Snell et al., 2021). Adults with psychiatric disorders, compared to those without such disorders, have higher rates of smoking and nicotine dependence, and lower rates of smoking cessation, which may be partly explained by the use of nicotine as a maladaptive coping strategy to regulate negative affect, given its effects on serotonergic and stress-related neurobiological systems (Grant et al., 2004; Lasser et al., 2015; Weinberger et al., 2017; Snell et al., 2021; Krebs et al., 2018; Goodwin et al., 2012). However, chronic nicotine use can exacerbate depressive and anxiety symptoms by altering neural circuits involved in stress regulation and increasing sensitivity to environmental stressors (Fluharty et al., 2017; Markou et al., 1998). In this context, affective distress does not act merely as a side effect, but as a key mechanism linking an individual’s basic biological vulnerability to the intensity of addiction itself. On the other hand, alcohol, as a central nervous system depressant, can induce feelings of sadness and irritability, even after moderate consumption (Schuckit & Monteiro, 1988). Chronic and heavy alcohol use is often accompanied by life problems, which further exacerbate depressive and anxiety symptoms. Individuals seeking treatment for alcohol dependence often have multiple concurrent symptoms, highlighting the complex relationship between substance use and affective disorders (Ross et al., 1990). According to the self-medication theory (Khantzian, 1997), people do not consume alcohol for hedonic reasons, but as an attempt to alleviate specific and intolerable affective states, with alcohol serving as a “chemical defense mechanism” to achieve temporary emotional stability (Radmilović et al., 2021). When talking about psychiatric patients, especially those with depression and anxiety disorders, the prevalence of smoking and alcohol problems is significant, and these phenomena are often interconnected with more severe symptoms of psychological difficulties (Mamić, 2015). For example, people with nicotine addiction have increased rates of depression and anxiety disorders compared to non-smokers, even after controlling for other risk factors (Grant et al., 2004). On the other hand, large epidemiological samples have shown that among people with nicotine addiction, many also have elevated rates of depression and anxiety, as well as alcohol use disorders (Breslau et al., 1991). Also, in studies involving people with diagnoses of depression and/or anxiety, the prevalence of alcohol dependence is several times higher than in control groups without these disorders (e.g., about 20% vs. 5% in controls), which indicates frequent comorbidity and mutual reinforcement of these conditions (Boschloo et al., 2011).

The relationship between anxiety, including specific phobic entities, and alcohol or drug addiction has been confirmed through previous research (Ross et al., 1990; Marks et al., 1966; Quitkin et al., 1972; Samarasinghe et al., 1984). It is assumed that anxiety states serve as ”transmitters” of the influence of more stable psychological constructs on the development of the addiction itself. It has been shown that people with anxiety disorders sometimes use alcohol as a form of self-medication to reduce phobic avoidance behavior and temporarily relieve anxiety (Cappell & Herman, 1972). However, excessive use of alcohol and other addictive substances can complicate clinical management, complicating the determination of the primary problem and the effective treatment of both disorders. These findings highlight the importance of studying the interrelationship of personality traits, anxiety/depression, and severity of addiction in patients with neurotic disorders.

Personality traits represent stable vulnerability or protective factors for the development of anxiety, depression, and addictive behaviors. Neuroticism is consistently associated with higher levels of affective symptoms, while agreeableness and conscientiousness are associated with lower levels of psychopathology (Lyon et al., 2021; T. Yang et al., 2023; S. W. Yang & Koo, 2022; Yasui-Furukori et al., 2019). However, personality traits are relatively stable and difficult to measure in direct clinical work, while anxiety and depression are fluctuating states that are often the immediate target of treatment. Therefore, it is important to explore the trajectory by which personality traits (as predispositions) through affective states (as mediators) lead to a more severe clinical picture of addiction. Despite extensive research, studies examining the mediating role of anxiety and depression in the relationship between personality traits and severity of alcohol and nicotine dependence, particularly in clinical populations with neurotic disorders (ICD-10: F40–F48), remain rare (Ross et al., 1990; Bjelland et al., 2002). Since the group of neurotic disorders encompasses a wide spectrum of diagnoses with different manifestations of anxiety and avoidance behaviors, it is necessary to examine how these specific differences within the clinical group modulate the relationship between personality and addiction. Furthermore, studies that simultaneously evaluate the mechanisms of alcohol and nicotine dependence within a single model are rare, thereby more clearly delineating the specific mediational pathways of anxiety and depression in the development of these often comorbid disorders.

Therefore, the aim of this study is to examine an integrative model in which anxiety and depression mediate the relationship between five main personality traits and the severity of alcohol and nicotine dependence. This approach will enable a more precise definition of whether the influence of personality on addiction is direct or whether it is primarily driven by the level of current affective distress in patients with neurotic disorders.

## 2. Materials and Methods

This study employed a cross-sectional design to examine the relationship between personality, affect, and addiction. A clinical sample of 232 adult patients was recruited from the General County Hospital Požega (OŽB Požega) over a two-year period, from April 2023 to April 2025. The study specifically targeted individuals with neurotic, stress-related, and somatoform disorders (ICD-10 codes F40–F48) who were capable of understanding the research objectives and providing written informed consent. To ensure a comprehensive representation of the neurotic spectrum, participants were recruited from multiple settings: psychiatric outpatient clinics, the day hospital, and the inpatient department. Participants from the inpatient setting were surveyed exclusively at the point of discharge, once clinical stabilization had been achieved, to ensure the absence of acute psychopathology or intoxication during assessment. The final sample included 133 (57.3) female and 99 (42.7) male participants, with a mean age of 48.58 years (SD = 10.77). A primary diagnosis of substance use disorders (F10–F19) served as an exclusion criterion.

### 2.1. Instruments

Big Five Inventory (BFI-44)—The BFI is used to assess personality traits, and consists of 44 items that respondents respond to on a five-point Likert scale, with 1 indicating “Strongly disagree” to 5 indicating “Strongly agree”. The scale consists of five subscales related to personality traits: extraversion (8 items, possible score range from 8 to 40), agreeableness (9 items, possible score range from 9 to 45), conscientiousness (9 items, possible score range from 9 to 45), neuroticism (8 items, possible score range from 8 to 40), and openness (10 items, possible score range from 10 to 50) (Mamić et al., 2024b; Costa Mastrascusa et al., 2023). The total score for each subscale is the average of all items related to a particular subscale. In this study, the reliability level of the BFI subscales, expressed as McDonald’s ω coefficient, showed high internal consistency for the neuroticism (ω = 0.89) and conscientiousness (ω = 0.84) subscales, indicating a strong relationship between questions within these personality dimensions. On the other hand, the agreeableness (ω = 0.70), extraversion (ω = 0.83), and openness (ω = 0.72) scales have a moderate level of reliability, suggesting some variability among questions within these dimensions, but still acceptable for research purposes.

Fagerström Test for Nicotine Dependence (FTND)—This scale serves to assess the physical component of nicotine dependence. The instrument consists of six items that measure key aspects of smoking behavior, primarily focusing on the daily amount of cigarettes consumed, the presence of compulsion, and the degree of dependence in situations that require abstinence. The total score is calculated by summing the responses on all particles, whereby a linear increase in the score correlates with a higher degree of physical dependence on nicotine, and can range from 0 to 10, with a higher number indicating a more severe nicotine dependence. The questionnaire is in the public domain and free for research use (Heatherton et al., 1991; Mamić et al., 2025). In this research, the reliability of the questionnaire, measured by McDonald’s ω coefficient, is 0.93, which represents an excellent level of internal consistency.

The Michigan Alcoholism Screening Test (MAST)—This test is an instrument designed to assess the severity of alcohol dependence through twenty-four items. The questions are answered in a dichotomous choice format (“yes” or “no”). Most affirmative answers are scored with one point, while items 1 and 4 are scored recoded, i.e., a point is awarded for a negative answer. The total score range is from 0 to 24, with higher values indicating more pronounced problems related to alcohol consumption (Ross et al., 1990; Mamić et al., 2024a). In this research, the instrument shows an excellent level of internal consistency with a McDonald’s ω coefficient of 0.94.

Depression, Anxiety, and Stress Scale (DASS-21)—This scale is used to assess three related negative emotional states. It consists of a total of 21 items, with each of the three subscales containing seven statements. Respondents assess the frequency and severity of symptoms during the past week on a Likert scale from 0 (did not apply to me at all) to 3 (did completely apply to me). The total score for each domain is the sum of all responses and can range from 0 to 21. Higher scores indicate greater symptom severity. The instrument is widely recognized for its excellent psychometric properties and high reliability across a variety of clinical and nonclinical populations (Lovibond & Lovibond, 1995). In this research, the internal consistency coefficients for the subscales are high, with a McDonald’s ω coefficient of 0.840 for depression and 0.910 for anxiety.

### 2.2. Statistical Methods

Data analyses were conducted using the JASP statistical package (version 19.3; JASP Team, 2024). First, descriptive analyses (means, standard deviations, frequencies, and percentages) were performed to present the basic characteristics of the sample and the distribution of scores on the scales used. In order to determine the normality of the distribution of variables, the Shapiro–Wilk test was used. Spearman’s correlations were used for a preliminary examination of the relationships between personality traits, defense mechanisms, and suicidality, because the conditions for conducting the parametric test were not met (normality of distribution). The main analysis was a mediation analysis.

We analyzed the relationship between personality traits (predictors) and the severity of alcohol and nicotine dependence (dependent variables), with affective states (depression and anxiety) included as potential mediators. The mediation model allowed us to distinguish between the direct effect (the direct influence of personality traits on the severity of dependence) and the indirect effect (the influence of personality traits on dependence via affective states).

To ensure the robustness of the results, the model was estimated using the Maximum Likelihood Robust (MLR) estimator. The MLR estimator was chosen because it provides standard errors and fit indices that are robust to non-normality in the data, which is common in clinical populations. Before conducting the analysis, all critical assumptions for regression and mediation modeling were verified. Multicollinearity was assessed using the Variance Inflation Factor (VIF), and the independence of residuals was tested via the Durbin–Watson (DW) statistic. Linearity of relationships and homoscedasticity was also confirmed. An indirect effect was considered significant if the 95% confidence interval did not include zero. The statistical significance level was set at *p* < 0.05, and all tests were two-tailed.

## 3. Results

Table 1 presents descriptive data on the sample of respondents who participated in the study. The total number of participants was 232, of which the majority were women (57.3), while men made up 42.7 of the sample. With regard to the clinical picture, the most common category were respondents with a diagnosis of F43 (Reactions to severe stress and adjustment disorders), which made up 30.2 of the sample, while the least common were those with a diagnosis of F48 (6.0). The average age of the respondents was 48.581 years (SD = 10.773) (Table 1).

Table 2 shows descriptive statistics and distribution parameters for the investigated variables. The average weight of alcohol addiction was M = 1.517 (SD = 3.936), while the average weight of smoking addiction was M = 2.164 (SD = 3.278). The analysis of the ot-wing distribution shows a marked positive asymmetry in the alcohol variable (Skewness = 2.827) with a very high flattening (Kurtosis = 7.216). Such results indicate that the majority of examinees are in the zone of low results, which is characteristic of zero inflation in clinical samples where addictive behavior is not present in all patients equally (Table 2).

Spearman’s correlation coefficients were used to determine the relationship between personality traits, affective states, and severity of alcohol and smoking addiction. The results indicate that the higher the levels of depression, the higher the levels of anxiety, with a strong positive association between these variables (ρ = 0.755; *p* < 0.001). Furthermore, the higher the level of neuroticism, the higher the levels of depression (ρ = 0.519; *p* < 0.001) and anxiety (ρ = 0.450; *p* < 0.001), as well as the severity of alcohol addiction (ρ = 0.250; *p* < 0.001) and smoking (ρ = 0.307; *p* < 0.001). The greater the severity of alcohol addiction, the more pronounced the levels of anxiety (ρ = 0.420; *p* < 0.001) and depression (ρ = 0.331; *p* < 0.001). Also, higher severity of alcohol dependence was associated with higher severity of smoking dependence (ρ = 0.273; *p* < 0.001). Similarly, the higher severity of smoking dependence, the higher levels of depression (ρ = 0.438; *p* < 0.001) and anxiety (ρ = 0.399; *p* < 0.001). On the other hand, higher levels of agreeableness were associated with lower levels of depression (ρ = −0.259; *p* < 0.001) and anxiety (ρ = −0.201; *p* = 0.002). Also, higher levels of conscientiousness were related to lower levels of depression (ρ = −0.346; *p* < 0.001), anxiety (ρ = −0.237; *p* < 0.001), and neuroticism (ρ = −0.191; *p* = 0.003). Finally, the higher the level of extraversion, the lower the level of depression (ρ = −0.284; *p* < 0.001), while extraversion was positively associated with conscientiousness (ρ = 0.415; *p* < 0.001) and openness (ρ = 0.294; *p* < 0.001) (Table 3).

The fit of the proposed structural model to the empirical data was assessed using several standard fit indices. The results showed an excellent, or rather complete, fit of the model to the data, with Comparative Fit Index (CFI) values of 1.000 and Tucker–Lewis Index (TLI) of 1.000. These values, which are well above the usual threshold of 0.950, indicate that the model perfectly explains the covariances among the examined variables. In addition, the Root Mean Square Error of Approximation (RMSEA) is <0.001, while the Standardized Root Mean Square Residual (SRMR) is also reduced to a value of <0.001. These results confirm that there is no significant deviation between the observed correlation matrix and the matrix predicted by the model.

**Figure 1:**
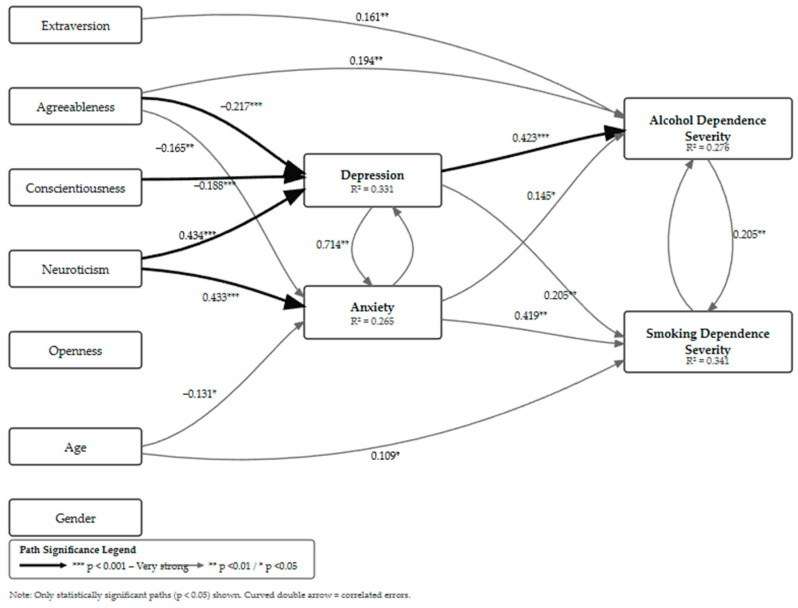
Schematic representation of the proposed mediation model of the relationship between personality traits, affective states, and addiction severity in patients with neurotic disorders, with only statistically significant standardized paths displayed (*N* = 232).

Such a high level of fit was achieved by using a robust MLR estimator, including demographic covariates (age and gender), and allowing for correlation between the residual errors of depression and anxiety. Residual linking was necessary due to the high correlation between these two mediators (r = 0.714, *p* ≤ 0.001), reflecting the common affective distress in patients with neurotic disorders. Including this correlation in the model ensured that the estimated pathways to addictive behaviors were cleared of methodological artifacts, thus meeting all criteria for reliable interpretation of direct and indirect effects in a clinical sample (Table 4) (Figure 1).

The results of the direct effects show that extraversion and agreeableness are statistically significant predictors of alcohol addiction severity. Extraversion (β = 0.161, *p* = 0.005) and agreeableness (β = 0.194, *p* = 0.003) were positively associated with alcohol addiction severity. For smoking addiction severity, extraversion showed a statistically significant direct effect (β = 0.145, *p* = 0.024), and age was also a significant predictor (β = 0.109, *p* = 0.035), while other personality traits did not reach the level of statistical significance (Table 5).

Indirect effects analysis showed that depression and anxiety did not generally mediate the relationship between personality traits and alcohol dependence severity, with a few important exceptions. Neuroticism had a significant indirect effect on alcohol dependence severity via anxiety (β = 0.181, *p* < 0.001), suggesting that higher levels of neuroticism contribute to higher levels of anxiety, which in turn contribute to higher alcohol dependence severity. Also, agreeableness had a significant negative indirect effect via anxiety (β = −0.078, *p* = 0.005), suggesting that lower anxiety associated with higher agreeableness contributes to lower alcohol dependence severity. For smoking dependence severity, neuroticism showed a significant indirect effect via depression (β = 0.183, *p* < 0.001), while the indirect effect via anxiety did not reach statistical significance. In addition, extraversion and agreeableness showed significant negative indirect effects via depression (β = −0.070, *p* = 0.020; β = −0.091, *p* = 0.004), suggesting that lower levels of depression associated with these traits contribute to lower severity of smoking dependence (Table 6).

The total effects for severity of alcohol dependence (ADS) show that extraversion (β = 0.161, *p* = 0.005) and agreeableness (β = 0.194, *p* = 0.003) are significant positive predictors. This indicates that higher levels of these traits are associated with greater severity of alcohol dependence in this sample. For severity of smoking dependence (SDS), significant total effects were observed for extraversion (β = 0.145, *p* = 0.024) and age (β = 0.109, *p* = 0.035). These results confirm that older age and higher extraversion are key total predictors of smoking addiction severity (Table 7).

The model explained 27.6% of the variance in the severity of alcohol dependence, 34.1% of the variance in the severity of smoking dependence, 33.1% of the variance in depression, and 26.5% of the variance in anxiety, indicating moderate explanatory power of the mediation model (Table 8).

## 4. Discussion

The results of the conducted research indicate a pronounced association between emotional states, personality dimensions and addictive behaviors within the studied clinical population (F40–F48). The strong positive correlation between depression and anxiety is particularly noteworthy, which confirms the high rate of comorbidity and overlapping symptoms within the spectrum of neurotic disorders. The results are consistent with previous research (Bjelland et al., 2002; Cannon & Weems, 2006; Morgan et al., 1998). Such a strong correlation suggests that anxiety and depressive symptoms can be considered variations of the same basic phenomenon that share a common psychological and biological predisposition (Jacobson & Newman, 2014).

Other results identified correlations that confirm the central role of neuroticism as a key dispositional factor that is positively related to both emotional states, but also to the severity of alcohol and nicotine addiction. Numerous previous studies have confirmed the aforementioned links, emphasizing neuroticism as a universal risk factor for the development of affective disorders (Yasui-Furukori et al., 2019; Lyon et al., 2021; S. W. Yang & Koo, 2022, Preetinanda et al., 2024; Leszko et al., 2020; Kotov et al., 2010). Since neuroticism, as a fundamental dimension of personality, usually precedes the appearance of symptoms, it is treated in the clinical literature as a basic risk factor that makes patients more susceptible to the development of anxiety and depression. The results are consistent with previous research that suggested that addictive behaviors in patients with neurotic disorders are not isolated entities, but are deeply rooted in their emotional status (Leszko et al., 2020). In part, these results confirm the theory of self-medication, according to which the consumption of psychoactive substances serves as an attempt to regulate unpleasant affective states (Khantzian, 1997).

Although correlations provide a broader insight into the connections between the examined constructs, they do not allow for a precise definition of the direction of action, which is crucial for understanding the relationship between personality traits, emotional states, and addiction severity. Therefore, structural equation modeling was used, which showed an excellent fit to the model, revealing that the process of personality influence on addictive behaviors in patients with neurotic disorders occurs through two separate mechanisms: direct and indirect. The analysis of direct effects indicated seemingly paradoxical paths that were not apparent from the previously mentioned correlations. Thus, it was shown that extraversion and agreeableness have a direct positive effect on the severity of alcohol addiction, while only extraversion has a positive effect on the severity of smoking addiction in patients with neuroses. Regarding extraversion, the results of previous studies, which were not conducted on this group of subjects, had mixed results. From how extraversion positively contributes to the severity of alcohol dependence (Turiano et al., 2012; Hicks et al., 2012) and smoking (Buczkowski et al., 2017) to the result that it has no effect on the severity of addiction (Preetinanda et al., 2024). Possible reasons for the results of this study are that people with higher extraversion traits are more sociable, talkative, and assertive (McCabe & Fleeson, 2012). Although in the general population extraversion can serve as a protective factor against social anxiety (Hassan et al., 2021), in patients with neurotic disorders, extraversion leads to greater social contacts, while simultaneous emotional instability can encourage the use of alcohol and nicotine as a coping strategy in social situations. The personality trait of agreeableness has also shown a positive direct effect on the severity of alcohol dependence. These findings are in contrast to previous results indicating that agreeableness has a protective effect on alcohol consumption (Franken & Prinzie, 2025; Kumari et al., 2023); however, these studies were not conducted on subjects with neurotic disorders. Possible reasons for such results are that people with a higher trait of agreeableness have a need to please others and maintain social relationships (Mamić et al., 2024b), which within the neurotic spectrum, where there is an increased fear of social rejection, may make them more sensitive to social pressure. In situations where alcohol consumption is a social norm, agreeableness in a neurotic patient may become a risk factor because alcohol is used as a means of social cohesion and avoidance of interpersonal conflicts.

It is also interesting that neuroticism, although expectedly correlated, did not show a direct effect on either alcohol dependence or smoking dependence in the model itself. Instead of a direct influence, neuroticism in patients with neuroses serves as a basis for the development of emotional states that become an immediate motive for substance consumption (Khantzian, 1997), which is in line with previous findings (Preetinanda et al., 2024; Leszko et al., 2020). This possibility is indicated by the findings of indirect effects, which showed that neuroticism affects the severity of alcohol dependence in a significant indirect way exclusively through anxiety, while it affects the severity of smoking dependence in a strong indirect effect primarily through depressive symptoms. Such mediation suggests that emotional instability in patients with neuroses must take a specific clinical form, either in the form of anxiety or depressive symptoms, in order to manifest as addictive behavior. The above results confirm the self-medication theory, according to which people with pronounced neurotic personality traits respond to stress by consuming alcohol or cigarettes to reduce negative emotions, but only if these emotions are intensely manifested (Kuntsche et al., 2005). Therefore, in patients with neurotic disorders, alcohol acts as an anxiolytic, while in smoking, the dominant influence of depression suggests that nicotine is used as a mechanism for regulating bad mood or anhedonia. Other significant results of indirect effects showed that extraversion and agreeableness act as significant protective factors, primarily through the reduction of negative affect. On the one hand, agreeableness reduces the severity of alcohol dependence through a lower level of anxiety symptoms and smoking through a reduction of depressive symptoms, while on the other hand, extraversion shows a significant negative indirect effect on the severity of smoking dependence through depressive symptoms. These findings are very interesting because at first glance they contradict previous findings of direct effects. While their direct effects suggest an increased risk for addiction, their indirect effects reveal a protective role. Namely, extraversion and agreeableness in patients with neurosis act as protective factors because they reduce the level of depressive affect and anxiety, thus eliminating the basic emotional incentive for alcohol and cigarette consumption. Extraversion and agreeableness as personality traits, when more pronounced, reduce tension and fear, but also bad mood (Luo et al., 2022), thus eliminating the possibility of the need for self-medication. These findings indicate a complex dynamic in which the same personality trait can act as both an incentive and a barrier to the development of addiction, depending on whether we observe its role in the social context or in the context of internal emotional regulation.

The total effects provided insight into the overall strength and direction of the effects of personality traits on the severity of addiction. The results confirmed that extraversion and agreeableness have significant positive total effects on alcohol addiction severity. In the case of alcohol, the role of these traits in facilitating social interactions and social cohesion predominates in the final contribution, despite their indirect protective role via distress reduction. For smoking, extraversion remained the only significant personality predictor at the total effect level. Interestingly, although neuroticism showed strong indirect pathways, its total effects on alcohol and smoking did not reach statistical significance in the final model, indicating that its influence is entirely channeled through specific affective states rather than acting as a simple additive risk factor in the total model. On the other hand, for smoking, the absence of an overall effect of agreeableness suggests that its protective role in regulating depressive affect is statistically neutralized by direct social factors.

The significant total effects for both types of addiction indicate that neuroticism is a universal risk factor in individuals with neurotic disorders. High and statistically significant total effects on both observed addictions indicate that neuroticism acts as a strong additive risk factor. It not only directly reduces the stress tolerance threshold, but also through the generation of specific affective states (anxiety and depression) creates a permanent need for external regulation of emotions. This finding suggests that high neuroticism in patients with neuroses may be a primary clinical indicator for a high risk of developing severe forms of addiction, which requires an integrated therapeutic approach that addresses both the basic personality and its symptomatic manifestations.

The values of the coefficients of determination indicate that the established mediation model has moderate to strong explanatory power. The percentage of explained variance for smoking addiction (34.1) and depressive symptoms (33.1) should be highlighted, which suggests that personality traits and their affective manifestation are fundamental factors in understanding these conditions. The model also explains a relevant percentage of the variance for anxiety symptoms (26.5) and alcohol dependence (27.6). These values indicate that there is still room for research on alcohol dependence and smoking in people with neurotic disorders, suggesting the existence of other factors that could moderate or further explain these relationships.

The results of the control variables included in the model, gender and age, partly clarified how demographic factors contribute to emotional distress, but also to the severity of addiction. Thus, it was shown that the age of the subjects is a significant direct predictor of the severity of smoking addiction, i.e., chronological age has an independent contribution to the intensity of smoking addiction in patients with neurotic disorders, regardless of their emotional status or personality traits. These results can be viewed as a cumulative effect of long-term nicotine exposure, which may lead to stronger neuroadaptations and biological dependence that are more difficult to modify than in younger individuals (Fagerström & Furberg, 2008).

Gender was shown to be a significant predictor of anxiety, indicating that gender differences play a role in the manifestation of anxiety symptoms within this clinical sample, with women reporting higher levels of anxiety, which is consistent with previous research (McLean et al., 2011). However, gender was not shown to play a significant role in directly contributing to the severity of alcohol and smoking dependence, confirming that the psychological mediation mechanisms identified by this model are relatively universal and stable regardless of the underlying demographic characteristics of the patients.

### 4.1. Practical Implications

The above results can be directly applied in practice through the design of interventions intended for patients with neurotic disorders. The focus in therapy should also be on emotional regulation skills, not just on abstinence. On the one hand, in patients with alcohol-dependent neurotic disorders, it is crucial to address anxiety management mechanisms, while in smokers, treatment must include strategies to alleviate depressive affect and anhedonia.

Furthermore, the recognized “dual role” of extraversion and agreeableness suggests the need for social skills and assertiveness training. Since these features, although emotionally protective of the patient, simultaneously increase the risk of succumbing to social pressure in environments where alcohol and nicotine are consumed, it is important to empower patients to maintain social contacts without endangering their own health. Finally, the high explained variance of the model justifies the introduction of a routine personality assessment (such as the Five-Factor Inventory) at the patient’s entry into treatment, so that the risk of developing severe forms of addiction could be predicted based on the profile of neuroticism and extraversion and preventive action could be taken in a timely manner.

### 4.2. Limitations of the Study

Despite the significant findings, this study has several limitations that should be taken into account when interpreting the results. First, the study is cross-sectional in nature. Although mediation analysis within the framework of structural equation modeling suggests a causal direction, there is the possibility of reverse causality, where long-term addiction may have a reciprocal effect on the level of depression or even on stable personality traits over time. Second, the study relied exclusively on self-report measures. In a clinical population, there is a risk of social desirability phenomena or cognitive biases in assessing one’s own emotional state and intensity of substance consumption. Third, the sample consisted exclusively of patients with diagnoses from the neurotic spectrum of disorders (F40–F48). Although this provided a deeper insight into this specific group, the results cannot be automatically generalized to the general population or to patients with other psychiatric disorders.

## 5. Conclusions

The conclusion of the study indicates that personality traits and emotional states are key determinants of the severity of addiction in patients with neurotic disorders, with neuroticism confirmed as the strongest universal risk factor. Its influence on both measured addictions is not direct, but is fully mediated by emotional states, which indicates that neuroticism serves as a dispositional basis that must take the form of a specific clinical symptom in order to manifest itself through addictive behavior. In this process, a high specificity of mediation pathways was determined, where neuroticism affects the severity of alcohol addiction exclusively through anxiety, while it affects smoking addiction through depressive symptoms, thus confirming the role of alcohol as an anxiolytic and nicotine as a means of regulating low affect.

The study also revealed the dual role of extraversion and agreeableness, which simultaneously act as risk factors in the direct social context, but also as significant protective factors through the reduction of anxiety and depression. The total effects confirmed that extraversion remains a significant overall predictor for both alcohol and smoking severity, suggesting that its role in social facilitation ultimately outweighs its protective affective influence in this clinical sample. The overall model showed moderate to strong explanatory power, accounting for 34.1 of the variance in smoking severity and 27.6 of the variance in alcohol severity, confirming that the integration of personality traits and affective states provides a relevant framework for understanding and treating addictive behaviors within the neurotic spectrum.

## Figures and Tables

**Table 1:** Sociodemographic characteristics and clinical profile of the participants (*N* = 232).

						*n* (%)
Gender	Female					133 (57.3)
	Male					99 (42.7)
Diagnosis	F40 Phobic anxiety disorders					45 (19.4)
	F41 Other anxiety disorders					55 (23.7)
	F42 Obsessive-compulsive disorder					24 (10.3)
	F43 Reaction to severe stress, and adjustment disorders					70 (30.2)
	F45 Somatoform disorders					24 (10.3)
	F48 Other neurotic disorders					14 (6)
Variable	Min	Max	M	SD	Skewness	Kurtosis
Dob	22	74	48.581	10.773	−0.281	−0.442

Note: Min—Minimum value; Max—Maximum value; M—Mean; SD—Standard Deviation.

**Table 2:** Descriptive statistics and correlations between personality traits, depression, anxiety, and severity of alcohol and smoking dependence (*N* = 232).

Variable	Min	Max	M	SD	Skewness	Kurtosis
Alcohol dependence severity	0	18	1.517	3.936	2.827	7.216
Smoking dependence severity	0	10	2.164	3.278	1.258	0.068
Depression	1	21	9.651	5.374	0.325	−0.733
Anxiety	0	21	9.677	4.968	0.362	−0.706
Extraversion	1.375	4.500	2.979	0.686	−0.065	−0.369
Agreeableness	1.889	4.444	3.323	0.472	−0.743	1.108
Conscientiousness	1.778	4.778	3.524	0.624	−0.761	0.601
Neuroticism	1	5.000	3.317	0.835	−0.354	0.101
Openness	2	4.400	2.947	0.461	0.602	1.346

Note: Min—Minimum value; Max—Maximum value; M—Mean; SD—Standard Deviation.

**Table 3:** Spearman correlation coefficients between personality traits, affective states, and severity of alcohol and smoking addiction (*N* = 232).

		2.	3.	4.	5.	6.	7.	8.	9.
1. Alcohol dependence severity	ρ	0.273	0.331	0.420	0.061	0.074	−0.072	0.250	0.016
	*p*	<0.001	<0.001	<0.001	0.355	0.263	0.277	<0.001	0.807
2. Smoking dependence severity	ρ		0.438	0.399	0.070	−0.089	−0.021	0.307	−0.047
	*p*		<0.001	<0.001	0.286	0.178	0.750	<0.001	0.480
3. Depression	ρ			0.755	−0.284	−0.259	−0.346	0.519	−0.091
	*p*			<0.001	<0.001	<0.001	<0.001	<0.001	0.166
4. Anxiety	ρ				−0.100	−0.201	−0.237	0.450	−0.073
	*p*				0.129	0.002	<0.001	<0.001	0.271
5. Extraversion	ρ					0.135	0.415	−0.121	0.294
	*p*					0.040	<0.001	0.066	<0.001
6. Agreeableness	ρ						0.307	−0.101	0.177
	*p*						<0.001	0.124	0.007
7. Conscientiousness	ρ							−0.191	0.082
	*p*							0.003	0.213
8. Neuroticism	ρ								−0.081
	*p*								0.220
9. Openness	ρ								-
	*p*								-

Note: *p*—Statistical significance; ρ—Spearman’s correlation coefficient.

**Table 4:** Model fit indices for the structural mediation model (*N* = 232).

Fit Index	Value
Comparative Fit Index (CFI)	1.000
Tucker–Lewis Index (TLI)	1.000
RMSEA	<0.001
SRMR	<0.001
Akaike Information Criterion (AIC)	4904.982
Bayesian Information Criterion (BIC)	5035.958

**Table 5:** Direct effects of personality traits on alcohol and smoking addiction severity (*N* = 232).

Predictor	→	Outcome	Estimate	SE	z-Value	*p*	95% CI Lower	95% CI Upper
Extraversion	→	ADS	0.161	0.058	2.798	0.005	0.048	0.274
Agreeableness	→	ADS	0.194	0.066	2.954	0.003	0.065	0.323
Conscientiousness	→	ADS	−0.079	0.059	−1.341	0.180	−0.194	0.036
Neuroticism	→	ADS	0.048	0.054	0.890	0.373	−0.058	0.155
Openness	→	ADS	0.017	0.067	0.256	0.798	−0.115	0.149
Age	→	ADS	0.030	0.056	0.539	0.590	−0.079	0.139
Gender	→	ADS	−0.069	0.052	−1.333	0.183	−0.170	0.032
Extraversion	→	SDS	0.145	0.064	2.250	0.024	0.019	0.271
Agreeableness	→	SDS	0.005	0.075	0.062	0.951	−0.143	0.152
Conscientiousness	→	SDS	0.049	0.060	0.825	0.409	−0.068	0.167
Neuroticism	→	SDS	0.081	0.063	1.290	0.197	−0.042	0.203
Openness	→	SDS	−0.071	0.061	−1.158	0.247	−0.192	0.049
Age	→	SDS	0.109	0.052	2.107	0.035	0.008	0.211
Gender	→	SDS	0.042	0.054	0.775	0.439	−0.064	0.148

Note: ADS—Alcohol dependence severity; SDS—Smoking dependence severity; SE—Standard error; CI—Confidence interval; *p*—Statistical significance.

**Table 6:** Indirect effects of personality traits on severity of alcohol and smoking dependence (*N* = 232).

Predictor	Mediator	Outcome	Estimate	SE	z-Value	*p*	95 CI Lower	95 CI Upper
Extraversion	Depr.	ADS	−0.011	0.019	−0.578	0.563	−0.048	0.026
Extraversion	Anx.	ADS	0.009	0.028	0.321	0.748	−0.045	0.063
Agreeableness	Depr.	ADS	−0.014	0.025	−0.560	0.575	−0.063	0.035
Agreeableness	Anx.	ADS	−0.078	0.028	−2.785	0.005	−0.133	−0.023
Conscientiousness	Depr.	ADS	−0.005	0.009	−0.555	0.578	−0.023	0.013
Conscientiousness	Anx.	ADS	−0.030	0.027	−1.111	0.266	−0.083	0.023
Neuroticism	Depr.	ADS	0.029	0.049	0.591	0.554	−0.067	0.125
Neuroticism	Anx.	ADS	0.181	0.042	4.310	<0.001	0.099	0.264
Openness	Depr.	ADS	0.005	0.011	0.454	0.649	−0.016	0.026
Openness	Anx.	ADS	0.013	0.026	0.500	0.617	−0.038	0.064
Extraversion	Depr.	SDS	−0.070	0.030	−2.333	0.020	−0.129	−0.011
Extraversion	Anx.	SDS	0.003	0.011	0.272	0.785	−0.018	0.024
Agreeableness	Depr.	SDS	−0.091	0.032	−2.844	0.004	−0.154	−0.028
Agreeableness	Anx.	SDS	−0.030	0.020	−1.500	0.133	−0.069	0.009
Conscientiousness	Depr.	SDS	−0.031	0.026	−1.192	0.233	−0.082	0.020
Conscientiousness	Anx.	SDS	−0.011	0.012	−0.916	0.359	−0.034	0.012
Neuroticism	Depr.	SDS	0.183	0.048	3.812	<0.001	0.089	0.277
Neuroticism	Anx.	SDS	0.069	0.040	1.725	0.084	−0.009	0.147
Openness	Depr.	SDS	0.036	0.026	1.384	0.166	−0.015	0.087
Openness	Anx.	SDS	0.005	0.010	0.500	0.617	−0.014	0.024

Note: Depr.—Depression; Anx.—Anxiety; ADS—Alcohol dependence severity; SDS—Smoking dependence severity; SE—Standard error; CI—Confidence interval; *p*—Statistical significance.

**Table 7:** Total effects of personality traits on the severity of alcohol and smoking dependence (*N* = 232).

Predictor	Outcome	Estimate (β)	SE	z-Value	*p*	95 CI Lower	95 CI Upper
Extraversion	ADS	0.161	0.058	2.798	0.005	0.048	0.274
Agreeableness	ADS	0.194	0.066	2.954	0.003	0.065	0.323
Conscientiousness	ADS	−0.079	0.059	−1.341	0.180	−0.194	0.036
Neuroticism	ADS	0.048	0.054	0.890	0.373	−0.058	0.155
Openness	ADS	0.017	0.067	0.256	0.798	−0.115	0.149
Age	ADS	0.030	0.056	0.539	0.590	−0.079	0.139
Gender	ADS	−0.069	0.052	−1.333	0.183	−0.170	0.032
Extraversion	SDS	0.145	0.064	2.250	0.024	0.019	0.271
Agreeableness	SDS	0.005	0.075	0.062	0.951	−0.143	0.152
Conscientiousness	SDS	0.049	0.060	0.825	0.409	−0.068	0.167
Neuroticism	SDS	0.081	0.063	1.290	0.197	−0.042	0.203
Openness	SDS	−0.071	0.061	−1.158	0.247	−0.192	0.049
Age	SDS	0.109	0.052	2.107	0.035	0.008	0.211
Gender	SDS	0.042	0.054	0.775	0.439	−0.064	0.148

Note: ADS—Alcohol dependence severity; SDS—Smoking dependence severity; SE—Standard error; CI—Confidence interval; *p*—Statistical significance.

**Table 8:** Explained variance (R^2^) of endogenous variables in the mediation model (*N* = 232).

Variable	R^2^
Alcohol dependence severity	0.276
Smoking dependence severity	0.341
Depression	0.331
Anxiety	0.265

Note: R^2^—Adjusted coefficient of determination.

## Data Availability

The data presented in this study are available on request from the first or corresponding authors.
